# Insights into the microbiological and virulence characteristics of bacteria in orthopaedic implant infections: A study from Pakistan

**DOI:** 10.1371/journal.pone.0292956

**Published:** 2023-10-17

**Authors:** Sidra Abbas, Azra Yasmin, Nouman Maqbool, Asim Ali Shah, Anila Fariq

**Affiliations:** 1 Microbiology and Biotechnology Research laboratory, Department of Biotechnology, Fatima Jinnah Women University, Rawalpindi, Pakistan; 2 Department of Orthopaedic Surgery, Fauji Foundation Hospital, Rawalpindi, Pakistan; 3 Microbiology Laboratory, Fauji Foundation Hospital, Rawalpindi, Pakistan; Government College University, Faisalabad, PAKISTAN

## Abstract

The exponential increase in the prevalence of multidrug resistant bacteria has resulted in limiting surgical treatment options globally, potentially causing biofilm-related complications, implant failure, and severe consequences. This study aims to isolate and characterize bacteria from post-surgical orthopaedic implant infections and screening for multiple antibiotic resistance. A cross-sectional study was conducted, involving isolation of forty-four dominant pathogenic bacterial isolates from 16 infected implant samples from across Islamabad and Rawalpindi. Out of forty-four, 38% cocci and 61% bacilli were obtained. Approximately 90% of isolates showed multiple antibiotic resistance (MAR) index of more than 0.2. Eleven strains were identified via 16S rRNA gene sequencing as *Pseudomonas aeruginosa*, *Bacillus* spp., *Planococcus chinensis*, *Staphylococcus*, *Escherichia coli* and *Enterobacter cloacae*. The bacterial strain *E*. *coli* MB641 showed sensitivity to Polymyxin only, and was resistant to all other antibiotics used. Maximum biofilm forming ability 0.532 ± 0.06, 0.55 ± 0.01 and 0.557 ± 0.07 was observed in *Pseudomonas aeruginosa* MB663, *Pseudomonas aeruginosa* MB664 and *Bacillus* spp. MB647 respectively after 24 hours of incubation. EPS production of bacterial strains was assessed, the polysaccharides and protein content of EPS were found to be in the range of 11–32 μg/ml and 2–10 μg/ml, respectively. Fourier transform infrared spectroscopic analysis of EPS showed the presence of carbohydrates, proteins, alkyl halides, and nucleic acids. X-ray diffraction analysis revealed crystalline structure of EPS extracted from biofilm forming bacteria. These findings suggest a high prevalence of antibiotic-resistant bacteria in orthopaedic implant-associated surgeries, highlighting the urgent need for ongoing monitoring and microorganism testing in infected implants.

## Introduction

Orthopaedic implants in recent years have revolutionized the treatment of health related issues such as osteoarthritis and other distressing problems, for example bone fractures [1, 2]. The rate of infections during surgical operations has increased despite the availability of better procedure options and strategies [3–5]. Several researchers suggested in their findings that the breach to skin barriers during the surgical operations, exposed the body to various bacterial infections [6–8]. After the insertion of the orthopaedic implant, the inevitable deposition of the host body proteins onto the implant device forms a favourable environment for the microorganisms to colonize and develop into biofilms [4, 9]. These biofilms on the orthopaedic implants protect the bacteria from antimicrobial treatments, such as antibiotics, therefore limiting treatment options, which further result in poor clinical outcomes [10, 11]. Once developed into biofilms, the bacteria are very difficult to treat because of the greater resistance to conventional antibiotics. These bacteria also further evade the host defence systems [12–15]. Such infections pose further pressure on health systems by prolonged stays in health-care facilities, increased prescribing of antimicrobial agents—such as antibiotics—and laboratory costs, amongst many others [16–20].

Of the bacterial species identified in the orthopaedic implant infections, the Gram-positive *Staphylococci* are the most prevalent species. Different studies showed that *Staphylococcus aureus* accounts for 20–30% of infections whereas the coagulase-negative *Staphylococci* are responsible for approximately 20–40% of surgical implant infections. Other less frequent microbes identified in the orthopaedic implant-associated infections include the Gram-positive cocci, such as *Streptococci* and *Enterococci*, as well as Gram-negative bacilli, for example *Pseudomonas aeruginosa* and the *Enterobacteriaceae* family [21–24].

This study aimed to isolate and characterize bacteria from post-surgical orthopaedic implant infections as well as assess antibiotic resistance profiles, explore the capacity for biofilm formation, and elucidate the role of extracellular polymeric substances (EPS) in shaping both antibiotic resistance and the development of biofilms.

## Materials and methods

### Ethical approval statement

The ethical review committee of the Fauji Foundation Hospital and Fatima Jinnah Women University have thoroughly reviewed the study. The committees did not find anything in the study which was against the ethical guidelines of biomedical research involving animals and human subjects.

Ethics reference number: FJWU/ EC/ 2020/23.

### Sample collection, isolation, and identification of strains

The samples for this cross-sectional study were collected from orthopaedic departments at three hospitals: Fauji Foundation Hospital and Pakistan Atomic Energy Commission Hospital in Rawalpindi, and Kulsum International Hospital in Islamabad. In this study, we focused only on the culturable bacterial diversity present in the collected samples, therefore excluding non-culturable diversity. When a patient with an infected orthopaedic implant was admitted, the hospital team notified researchers of the surgery schedule. Surgeons provided discarded skin, deep tissue, and explanted prosthesis samples during surgery. These samples were immediately transferred to sterilized tubes containing 0.9% saline and then to the Microbiology and Biotechnology Research Laboratory at Fatima Jinnah Women University within an hour. The samples were then inoculated into brain heart infusion (BHI) broth, and grown aerobically for 24 hours at 37°C. Serial dilutions of the culture were prepared, and single bacterial colonies were obtained following serial dilution and streaking. The pure culture was preserved in Luria Bertani (LB) glycerol stock solution at -80°C. Overall, 44 distinct colonies were isolated, the morphology of the isolated colonies was studied with the naked eye as well as under microscopes. Gram staining procedure was applied to study the cell morphology and then biochemical characterization was carried out using API 10 S strips [25].

### Antibiotic susceptibility testing

Disc diffusion method proposed by Bauer and colleagues that involves placing a disk impregnated with a specific antibiotic on a Mueller Hinton agar plate containing the bacteria was used [26]. The bacteria was allowed to grow for 24 hours at 37 °C, and the zone of inhibition (the area around the disk where no bacteria are growing) was measured. The antibiotic discs (Oxoid, Hampshire, England) were purchased and used as directed by the manufacturer. The bacterial isolates were tested against ampicillin (30 μg), bacitracin (10 μg), cefuroxime (30 μg), chloramphenicol (30 μg), ciprofloxacin (5 μg), clindamycin (2 μg), rifampicin (5 μg), streptomycin (30 μg), sulfamethoxazole-trimethoprim (25 μg), tetracycline (30 μg), vancomycin (30 μg), polymyxin (300 μg), cotrimoxazole (25 μg), cephradine (25 μg), penicillin (10 μg), erythromycin (15 μg), methicillin (30 μg), augmentin (30 μg), imipenem (10 μg), doxycycline (30 μg), gentamycin (10 μg), cefotaxime (30 μg), and ceftriaxone (30 μg). The inhibition zone diameter was interpreted using clinical and laboratory standard institute (CLSI) guidelines [27]. The results were interpreted as sensitive, intermediate, and resistant.

#### Multiple antibiotic resistance (MAR) index calculation

The multiple antibiotic resistance (MAR) index was calculated for each isolate using the formula MAR = a/b, where ’a’ is the number of antibiotics to which the test isolate shown resistance and ’b’ is the total number of antibiotics to which the test isolate has been assessed for susceptibility.

### 16S rRNA gene sequencing analysis

The 16S rRNA gene, approximately 1500 bp long with 9 hypervariable regions (V1 –V9) was used for the identification of eleven strains [28]. The bacterial isolates were identified using the V3 and V4 hypervariable regions in the 16S rRNA gene. Genomic DNA was extracted from the bacteria following the protocol described by Vingataramin and Frost [29]. Colony PCR was carried out to amplify the 16S rRNA-encoding gene. Briefly, 250 ng of bacterial DNA was used as a template with 0.5 μM (each) primer (341F [5-CCTAYGGGRBGCASCAG-3] and 806R [5-GGACTACNNGGGTATCTAAT-3]), 200 μM deoxynucleoside triphosphate, and 2 U of *Taq* high-fidelity DNA polymerase (Boehringer Mannheim) in a 1× amplification buffer (10 mM Tris-HCl [pH 8.3], 50 mM KCl, 1.5 mM MgCl_2_). The PCR conditions consisted of denaturation of the mixture at 94°C for 2 minutes, the mixture was then subjected to 40 cycles of annealing at 56°C for 1 minute, 1 min of elongation at 72°C, and 1 min of denaturation at 94°C. A final extension step was achieved at 72°C for 10 minutes. PCR products were resolved by electrophoresis in a 1% agarose gel stained with ethidium bromide. The sequences obtained were verified online using the NCBI gene bank, http://www.ncbi.nlm.nih.gov/. After obtaining accession numbers, MEGA X software was used to generate a phylogenetic tree.

### Production and extraction of extracellular polymeric substances (EPS)

Fresh culture of bacterial strains was used for extraction of the EPS. The production of extracellular polymeric substances was carried out using EPS medium [30]. Flasks containing 50 mL of the EPS broth were inoculated with 1 mL of the bacterial suspension. Flasks were placed in a shaking incubator at 150 rev min^-1^, at 37 °C for 72 hour. The cells were collected through sedimentation (10,000 rev min-1) at 4 °C and for 15 min. The extracted EPS was obtained from the subsequent supernatant, which was precipitated using prechilled acetone (1:2) and kept at 4°C overnight. After spinning the mixture down at 10,000 rev min-1 for 15 min, the collected pellets were dried in a desiccator for further analysis.

#### Quantification and characterization of EPS

Protein content in the EPS was quantified as per the Bradford method [31]. The phenol sulfuric acid method was used for the quantification of total polysaccharides in the EPS [32]. Absorption was measured at 490 nm and the concentrations were determined using a standard curve of glucose. The main functional groups present in the EPS of the selected bacterial were identified via Fourier transform infrared spectroscopy (FTIR-8400 Schimadzu). Briefly, dried EPS samples were mixed with spectroscopic grade potassium bromide (KBr) in a 5:95 ratio. Pellets of the samples were prepared using a hydraulic presser. Infrared spectrum in the 4000–400 cm^-1^ region with 15 scan speed was generated using a spectrophotometer [33]. X-ray diffraction (XRD) of the powdered EPS was performed using copper-Kα x-rays at a wavelength of 1.5406 Å. 2θ data was obtained at a range from ~5° to 80°, with a scan step size of 0.02° step and time per step of 0.4 s with voltage of 40 Kv and 30 mA beam current [34].

### Bacterial cell adhesion to the hydrocarbon (BATH) test

Hydrophobic surface characteristics of bacterial cells were determined using the BATH test [35]. Briefly, the bacterial cells were cultured until the logarithmic phase in nutrient broth. The cells were pelleted and washed twice with a phosphate urea magnesium (PUM) buffer. The bacterial cells, normalized to an OD_400_ of 1.0 (1.2 mL), were transferred to a series of clean test tubes, to which hexadecane was added. The mixture in the test tubes was thoroughly mixed for 15 minutes. The test tubes were then left for 2 minutes for phase separation. Optical density of the aqueous phase was measured at 400 nm using an UV-visible spectrophotometer. PUM buffer was used as reference.

### Microtiter plate assay

Microtiter plate assay was used to observe the biofilm forming ability of bacterial strains [36]. An overnight culture was pelleted and washed with phosphate buffered saline (PBS). The culture suspension was then diluted to an OD_600_ of 0.05. An aliquot (100 μL volume) of the diluted culture was added to each well in a 96-well microtiter plate. The plate was then sealed with a sterile gas-permeable film and incubated without shaking at 37°C for 24 hours. After this time, the attached biomass was quantified by crystal violet staining. Briefly, the culture in each well was removed by aspiration. The wells were washed three times with deionized water (200 μL) and air-dried. The attached biomass was then stained by the addition of 0.1% w/v crystal violet for 10 min. Following this, the stain was removed by aspiration and the wells were washed three times with deionized water (200 μL) and air-dried. The residual crystal violet stain associated with the attached biomass was solubilized by adding 200 μL of acetic acid (30% v/v) in each well. After 10 minutes the OD_595_ was measured spectrophotometrically. A well containing sterile LB served as a negative control.

### Statistical analysis

Results of each experiment were stated as a mean of three technical replicate ± standard deviation (SD). One-way ANOVA with Fisher Pairwise Comparison was used for evaluating the biofilm formation capacity, extracellular polymeric substance production, and adhesion of the bacterial cell to hydrocarbon. Correlation analysis of hydrophobicity of the bacteria and EPS formation was determined by Principal Component Analysis (PCA) using Minitab 19 software. The clades were formed based on the similarity of biochemical tests. Strains having similar results were clustered together using PAST software version 3.12.

## Results

### Patient history and Sample collection

Samples were collected from 16 patients between 11–83 years old over the period of 1 year from different hospitals in Islamabad and Rawalpindi. All the patients were identified with post-operative infections. Risk factors that are associated with orthopaedic implant infections, include diabetes, hypertension, obesity, and older age. Patients with diabetes are at a higher risk of infection compared to other health related issues. The results indicated that 31.25% of the patients admitted for the revision surgery had either diabetes or pre-diabetes, whereas 18.75% had hypertension. The meta data of the clinical bacterial isolates obtained from discarded skin and a deep tissue samples as well as infected explanted prostheses provided by the operating surgeons is shown in Table 1.

**Table 1:** Metadata of the bacterial isolates.

Patients	Age/Sex	Bacterial Isolates	Isolation source	Disease history	Treatment
**1**	11/Male	**MB631**	Epidermis	None	Metal rod removed from left Ulna
		**MB632**	Implant		
		**MB633**	Deep tissue		
**2**	59/Male	**MB634**	Epidermis	None	Metal rod removed from right Ulna
		**MB635**	Deep tissue		
		**MB636**	Implant		
**3**	69/Male	**MB637**	Deep tissue	Hypertension/ Diabetes	Proximal femoral nail implant removal
		**MB638**	Implant		
		**MB639**	Implant		
**4**	65/Female	**MB640**	Epidermis	Diabetes/ obesity	2nd Knee arthroplasty
		**MB641**	Deep tissue		
		**MB642**	Epidermis		
**5**	57/Female	**MB643**	Deep tissue	Rheumatoid arthritis	Knee arthroplasty
		**MB644**	Implant		
		**MB645**	Epidermis		
**6**	42/Female	**MB646**	Deep tissue	None	Proximal femoral nail implant removal
		**MB647**	Deep tissue		
		**MB648**	Implant		
**7**	67/Female	**MB649**	Deep tissue	Osteoarthritis	Humeral nail implant removal
		**MB650**	Implant		
		**MB651**	Epidermis		
		**MB652**	Deep tissue		
**8**	52/Male	**MB653**	Deep tissue	None	Humeral nail implant removal
		**MB654**	Implant		
**9**	47/Male	**MB655**	Implant	None	Tibial intramedullary nail implant removal
		**MB656**	Epidermis		
		**MB657**	Deep tissue		
**10**	43/Female	**MB658**	Implant	None	Femoral nail implant removal
		**MB659**	Epidermis		
		**MB660**	Implant		
**11**	83/Female	**MB661**	Implant	Diabetes/ hypertension	Nail implant removal
		**MB662**	Deep tissue		
		**MB663**	Deep tissue		
**12**	38/Female	**MB664**	Deep tissue	Chest infection	Nail implant removal
		**MB665**	Epidermis		
		**MB666**	Epidermis		
**13**	75/Male	**MB667**	Implant	Diabetes/ obesity	Metal rod removed from right Ulna
		**MB668**	Implant		
**14**	50/Male	**MB669**	Deep tissue	Pre-diabetes	Nail implant removal
		**MB670**	Deep tissue		
**15**	51/Male	**MB671**	Epidermis	Hypertension	Proximal femoral nail implant removal
		**MB672**	Epidermis		
**16**	34/Male	**MB673**	Implant	None	Femoral nail implant removal
		**MB674**	Implant		

### Isolation of strains and morphological characteristics

Colony morphology of bacterial isolates was examined in terms of colony appearance. All colonies were circular in shape, having raised elevation and smooth margins, except for MB647, which had a wrinkled appearance. Out of forty-four isolates, seventeen bacterial isolates were cocci, whereas the rest belonged to group bacilli (S1 Table). Most strains were capsule formers. Cells of different isolates exhibited various arrangements, while some bacteria formed filaments (S2 Table). All the isolates were mesophiles and showed growth at the range of pH 7–9. Isolates MB633, MB635, MB637, MB638, MB663, MB664, MB665, MB666, MB667 and MB668 had mucilaginous, while the rest had a creamy texture.

### Biochemical characterization

**Fig 1:**
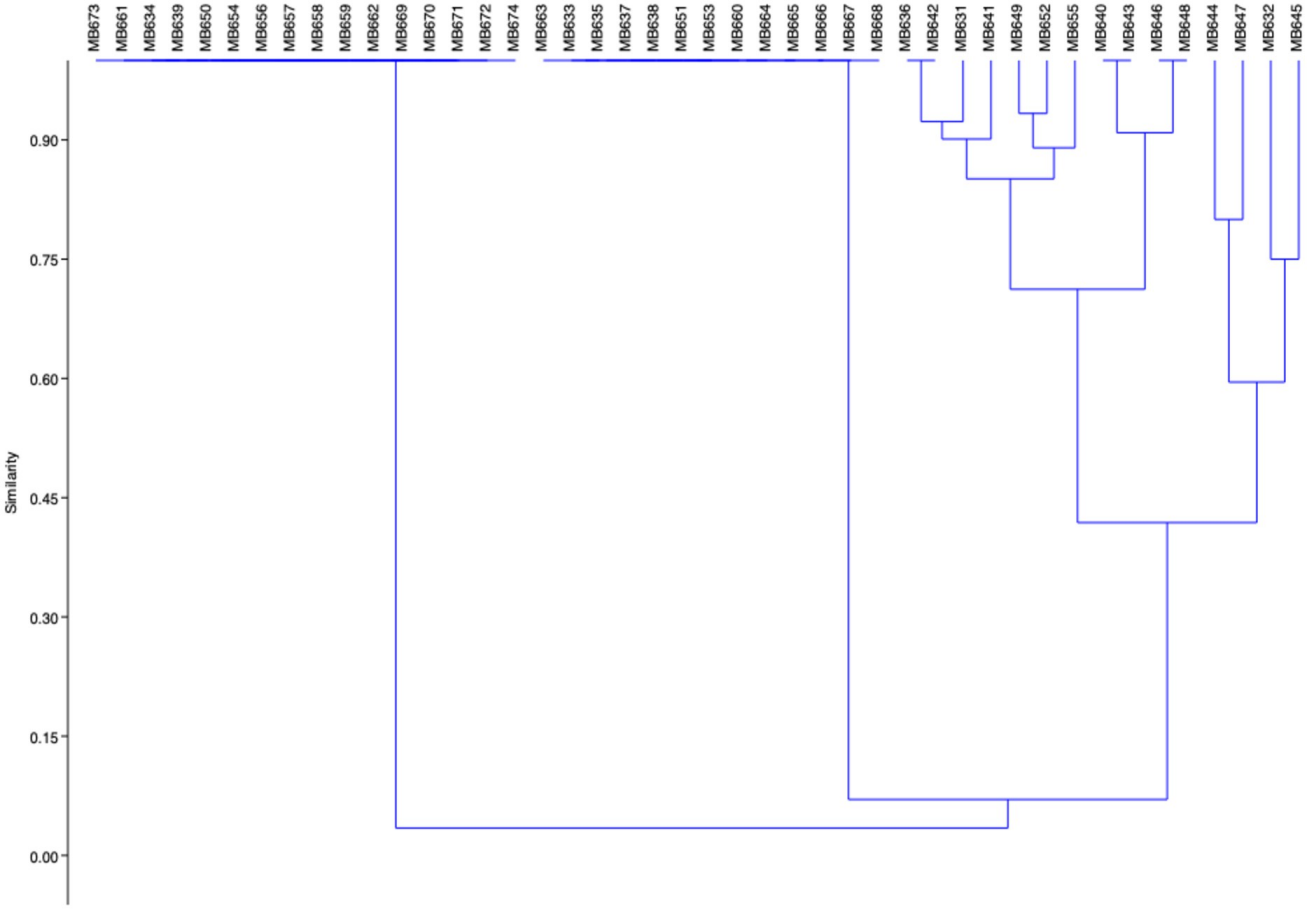
Clustering of the bacterial isolates obtained from the infected tissue samples based on various biochemical characters. Similarity among isolates was assessed by conducting 10 different biochemical sugar fermentation tests using the API 10 S strips and converting them into binary data, 0 for negative and 1 for positive test results, respectively, using PAST (Paleontological Statistics Software Package for Education and Data Analysis software). Similarities amongst the strains were estimated using the Jacquard coefficient, and the unweighted average linkage gave the cluster. Consequently, bacterial isolates sharing the greatest similarity are clustered together, enabling a clear visualization of their relatedness.

It was observed that most of the isolates were unable to utilize different sugars (S3 and S4 Tables). Few bacterial isolates (MB631, MB636, MB649, MB652, MB640, MB641, MB642, MB643, MB646 and MB648) were able to utilize D-glucose, nitrate reductase, indole and 2-nitrophényl-ßDgalactopyranoside. Only one isolate, MB642, showed hydrogen sulphide gas production. Isolates were identified and clustered into different groups based on phenotypical and biochemical characterization. Bacterial isolates MB634, MB636, MB639, MB650, MB654, MB656, MB657, MB658, MB659, MB661 MB662, MB669, MB670, MB671, MB672, MB673 and MB674 were catalase, Coagulase and DNase positive (Fig 1). Initially, bacterial isolates were identified through morphological and biochemical characteristics. Subsequent analysis of colony morphology and biochemical traits revealed significant strains including *Klebsiella pneumoniae*, *Pseudomonas* spp, *Bacillus* spp, *Staphylococcus aureus*, and *E*. *coli*. Isolates MB672, MB656, MB634, MB639, MB650, MB654, MB657, MB658, MB659, MB661, MB669, MB670, MB671 and MB674 were exactly similar to each other and identified as *Staphylococcus aureus* species. Isolates MB663, MB638, MB633, MB635, MB637, MB651, MB653, MB664, MB665, MB666, MB667 and MB668 are clustered together in a single clade and identified as *Pseudomonas* species. Cluster containing isolates MB640, MB643, MB646 and MB648 were identified as *E*. *coli*. Isolates MB649, MB652 and MB655 were grouped together and identified as *Klebsiella* on the basis of biochemical characterization. Isolates MB636, MB631, MB641 and MB642 were grouped together in a single cluster. Isolates MB636 and MB631 were identified as *Klebsiella* and MB642 and MB641 were identified as *E*. *coli* on the basis of biochemical characters.

### Antibiotic resistance profile

Bacterial strains showed resistance to most antibiotics used in the study. Bacterial isolates MB641 showed sensitivity to polymyxin only, and exhibited resistance to all other antibiotics used. Bacterial isolates MB645, MB647, MB650 and MB653 exhibited sensitivity towards sulfamethoxazole-trimethoprim, while the rest of the forty strains were resistant to this antibiotic. Isolates MB640, MB641, MB642, MB643, MB646 and MB648 showed resistance against ampicillin, cotrimoxazole, gentamycin, ciprofloxacin, vancomycin, ceftazidime, and ceftriaxone. Bacterial strains MB654, MB669, MB670, MB671, MB672, MB673, MB674 exhibited resistance against penicillin, erythromycin, methicillin, augmentin, cephradine, and imipenem. Only six bacterial isolates MB632, MB644, MB645, MB647, MB651 and MB660 were sensitive to tetracycline. MB640, MB642, MB643, MB646, MB648, MB650, MB656, MB658 were resistant to ciprofloxacin while the rest of the strains were sensitive to ciprofloxacin.

#### Multiple antibiotic resistance (MAR) index

**Fig 2:**
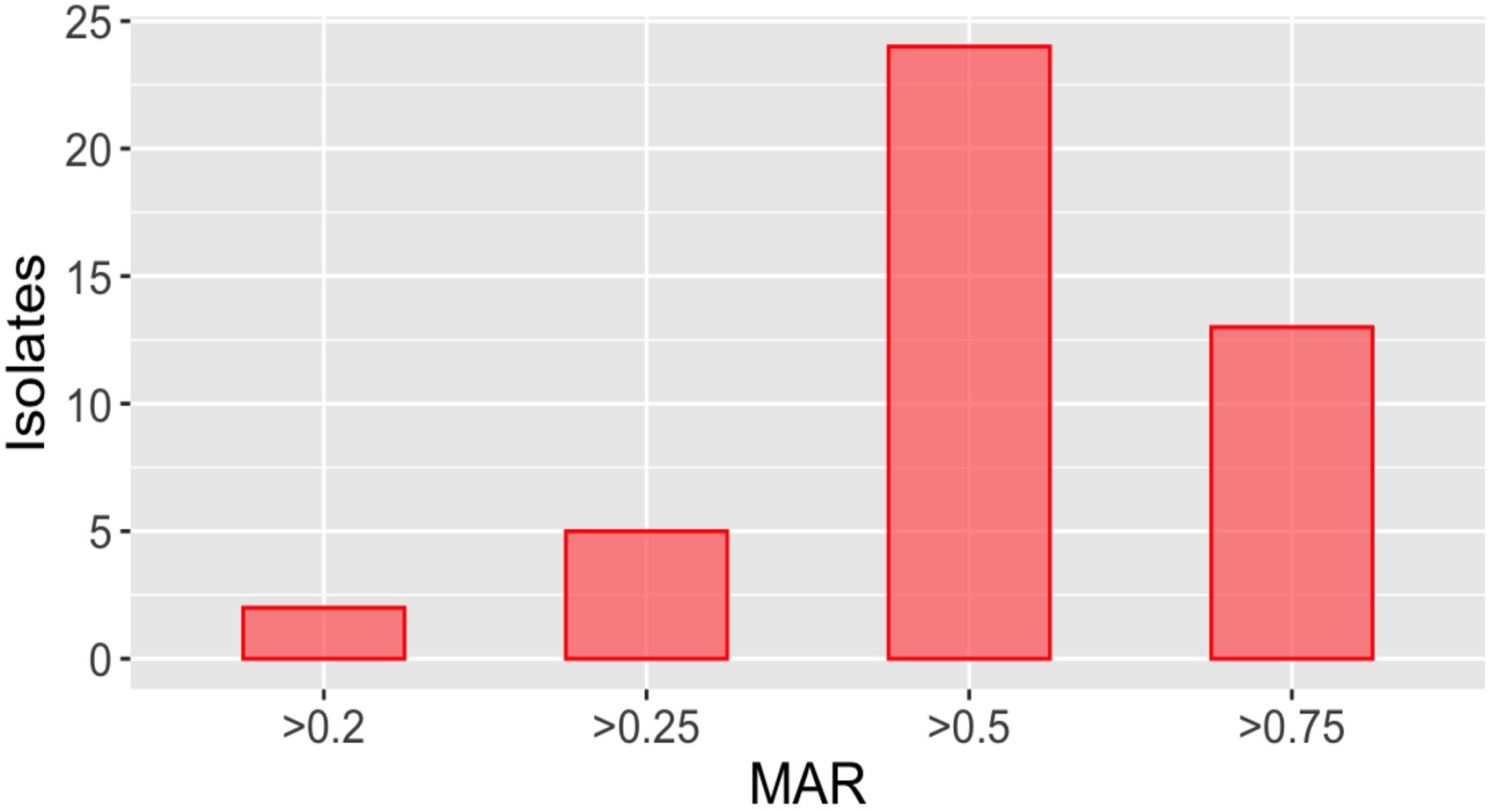
The graph depicting the bacterial species’ MAR index from samples of infected tissues. The MAR index is indicative of the level of resistance of the isolated bacterial isolates. The multiple antibiotic resistance (MAR) indices were determined with reference to the tested antibiotics, and it was above 0.2 in almost 90% of the bacteria studied. Highest MAR index was observed in MB655 (0.88) and MB649 (0.8) while lowest MAR index was observed in bacterial strain MB645 (0.14) and MB651 (0.15).

Strains were classified as sensitive, intermediate, and resistant to antibiotics, based on the size of the zone of inhibition. Multiple antibiotic resistance (MAR) computed for each strain showed high values. Multiple antibiotic resistance pattern in bacterial strains isolated from surgical implant infections was also studied. The majority of the bacterial strains exhibited resistance to the selected antibiotics. The overall trend indicated that MB642 and MB655 were the most resistant bacteria with a MAR index of 0.9, whereas MB643, MB646 and MB652 also exhibited multiple resistance against approximately 80% of antibiotics used, with a MAR index of 0.88. Isolates MB648, MB649, MB631, MB636, MB656, MB669, MB654, MB658 had a MAR index of 0.7. Isolates MB659, MB657, MB660, and MB661 showed a MAR index of 0.6. The lowest MAR index of 0.18 was observed in MB645 and MB651 (Fig 2).

### 16S rRNA gene sequencing analysis

**Fig 3:**
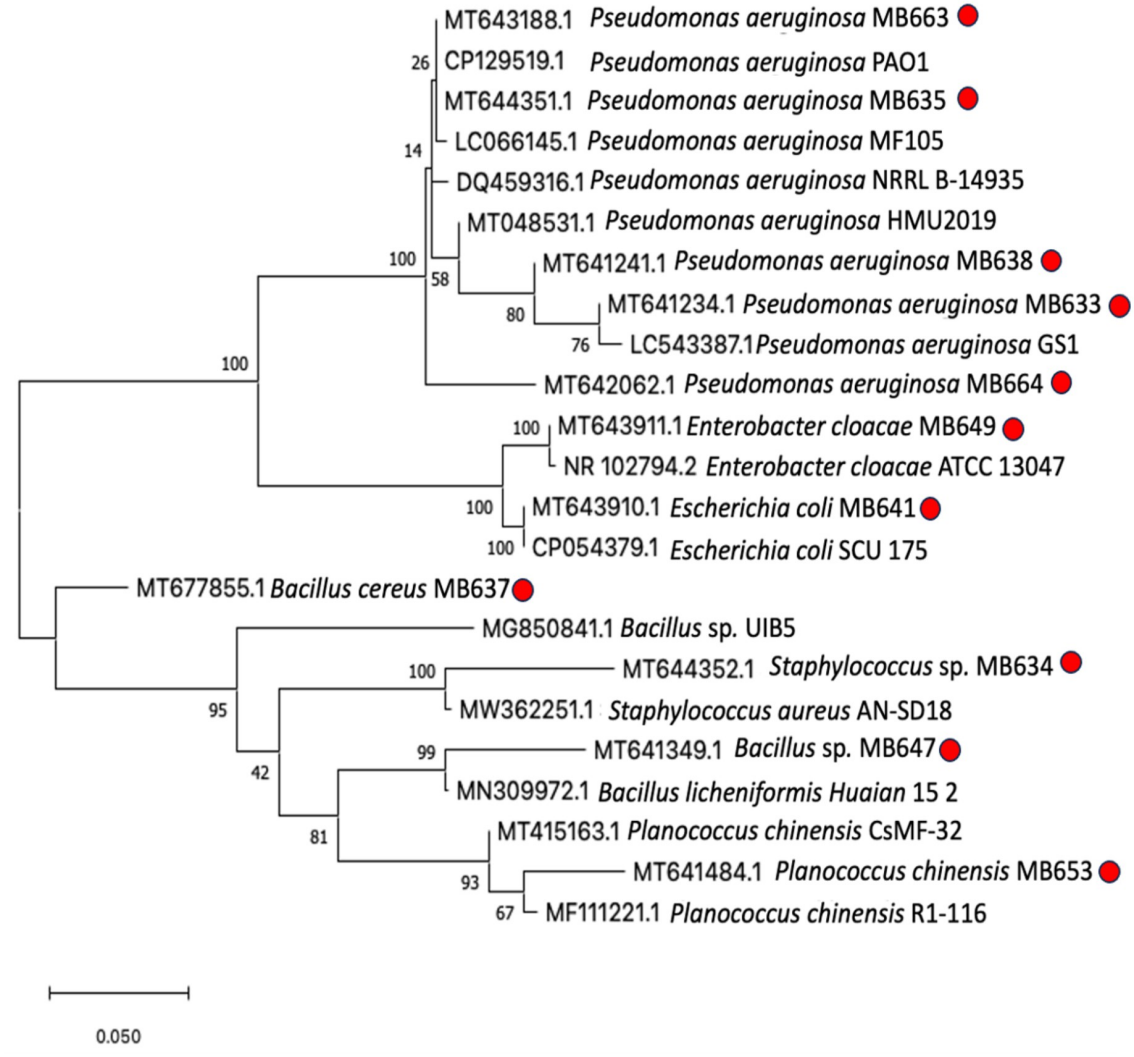
Phylogenetic tree of dominant bacterial diversity isolated from infected soft tissues. Evolutionary trees were constructed in MEGAX software by using Neighbor-Joining (NJ) method. Bootstrap values (expressed as percentages of 1000 replications) are shown at the branch points. Clinical isolates studied in present research are highlighted with a red dot. Eleven morphological distinct strains were selected, and the sequences obtained were aligned with their closest reference bacterial sequences available in GenBank using Basic Local Alignment Search Tool (BLAST) for identification indicated in the figure mentioned above.

After initial screening, including morphological, biochemical, and antibiotic resistance profiling, 11 bacterial strains were selected for 16S rRNA gene sequencing and further study. The identity of the screened strains was established using 16S rRNA gene sequencing and subsequent comparison of the output sequence data against the NCBI database http://www.ncbi.nlm.nih.gov/. The query cover, percentage identity, and closest match information for each strain can be found in Table 2 along with the corresponding accession numbers generated during the analysis. This information provides a comprehensive overview of the taxonomic classification and closest matches of the selected strains in our study. Phylogenetic analysis was carried out to determine the similarity with other bacterial strains of the same genus (Fig 3).

**Table 2:** NCBI accession numbers.

Strains	Accession numbers	Sequence length (bp)	Query cover (%)	% identify	Closest GenBank match
*Pseudomonas aeruginosa* MB633	MT641234	264	98	98.46	*P*. *aeruginosa* strain GS1
*Staphylococcus* MB634	MT644352	447	91	97.56	*Staphylococcus aureus* strain C182
*Pseudomonas aeruginosa* MB635	MT644351	1,541	100	100	*P*. *aeruginosa* strain PAO1
*Bacillus cereus* MB637	MT677855	255	98	93	*Bacillus sp*. strain UIB5
*Pseudomonas aeruginosa* MB638	MT641241	264	99	98.47	*P*. *aeruginosa* strain HMU2019
*Escherichia coli* MB641	MT643910	1,547	100	100	*Escherichia coli* strain SCU-175
*Bacillus* spp MB647	MT641349	445	95	98.13	*Bacillus licheniformis* strain Huaian_15_2
*Enterobacter cloacae* MB649	MT643911	588	100	99.83	*Enterobacter cloacae* strain ATCC 13047
*Planococcus chinensis* MB653 (basionym: *Planomicrobium* *chinense*)	MT641484	271	94	98.45	*Planococcus chinensis strain* R1-116
*Pseudomonas aeruginosa* MB663	MT643188	1,541	100	100	*P*. *aeruginosa* PA14
*Pseudomonas aeruginosa* MB664	MT642062	440	98	97.03	*P*. *aeruginosa* strain MF105

### Correlation between EPS production and hydrophobicity

**Fig 4:**
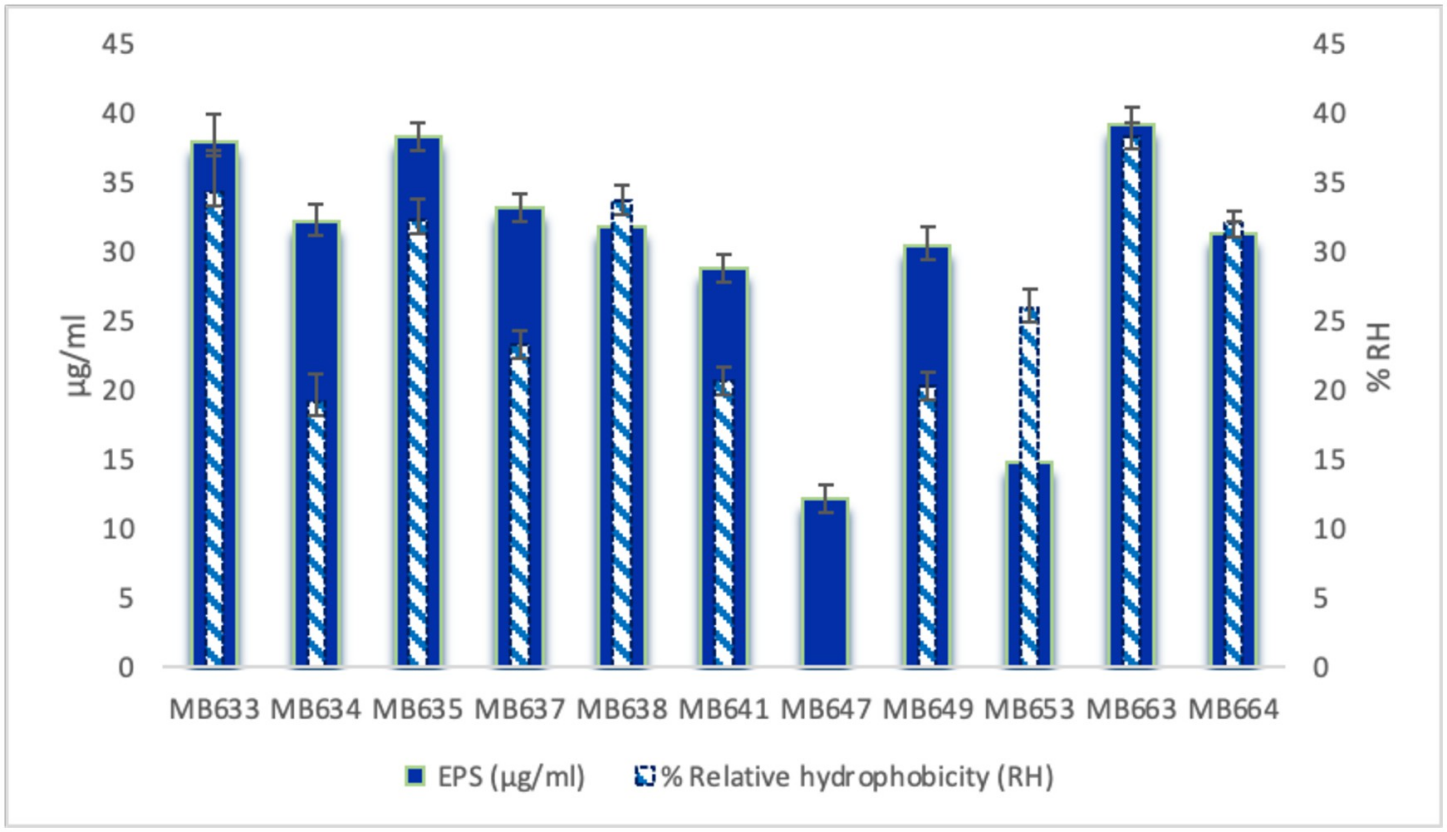
Correlation between the Extracellular polymeric substances production and hydrophobicity of selected isolates. The release of extracellular polymeric substance and relative hydrophobicity of the clinical isolates after 24 hours of incubation is shown in the figure above. A positive correlation can be observed between most strains. Strain 647 showed no tendency to adhesion toward hydrocarbon. Depicting the inability of the strain for adherence to hydrophobic surface. Data represents average of three technical replicates ± SD.

The EPS production and relative hydrophobicity of the bacterial strains was assessed. The polysaccharides and protein content of the EPS were found to be in the range of 11–32 μg/ml and 2–10 μg/ml, respectively. Correlation between the EPS production and hydrophobicity in clinical isolates was assessed after 24 hours of incubation. Results indicated a positive correlation between hydrophobicity and EPS production except in *Bacillus* spp. MB647 that showed no tendency towards hydrocarbons adhesion. *Bacillus* spp. MB647 also produced less extracellular polymeric substances as compared to other bacterial strains but highest biofilm formation ability. The highest hydrophobicity was observed in five *Pseudomonas* strains i.e., MB633, MB635, MB638, MB663 and MB664 (Fig 4), thereby showing their ability to adhere to the hydrocarbons. *Planococcus chinensis* MB653 also showed significant adhesion towards hydrocarbons. The principal component plot depicted a moderate correlation between extracellular polymeric substance and hydrophobicity (r = 0.630, *p* = 0.000).

### Identification of the functional groups

**Fig 5:**
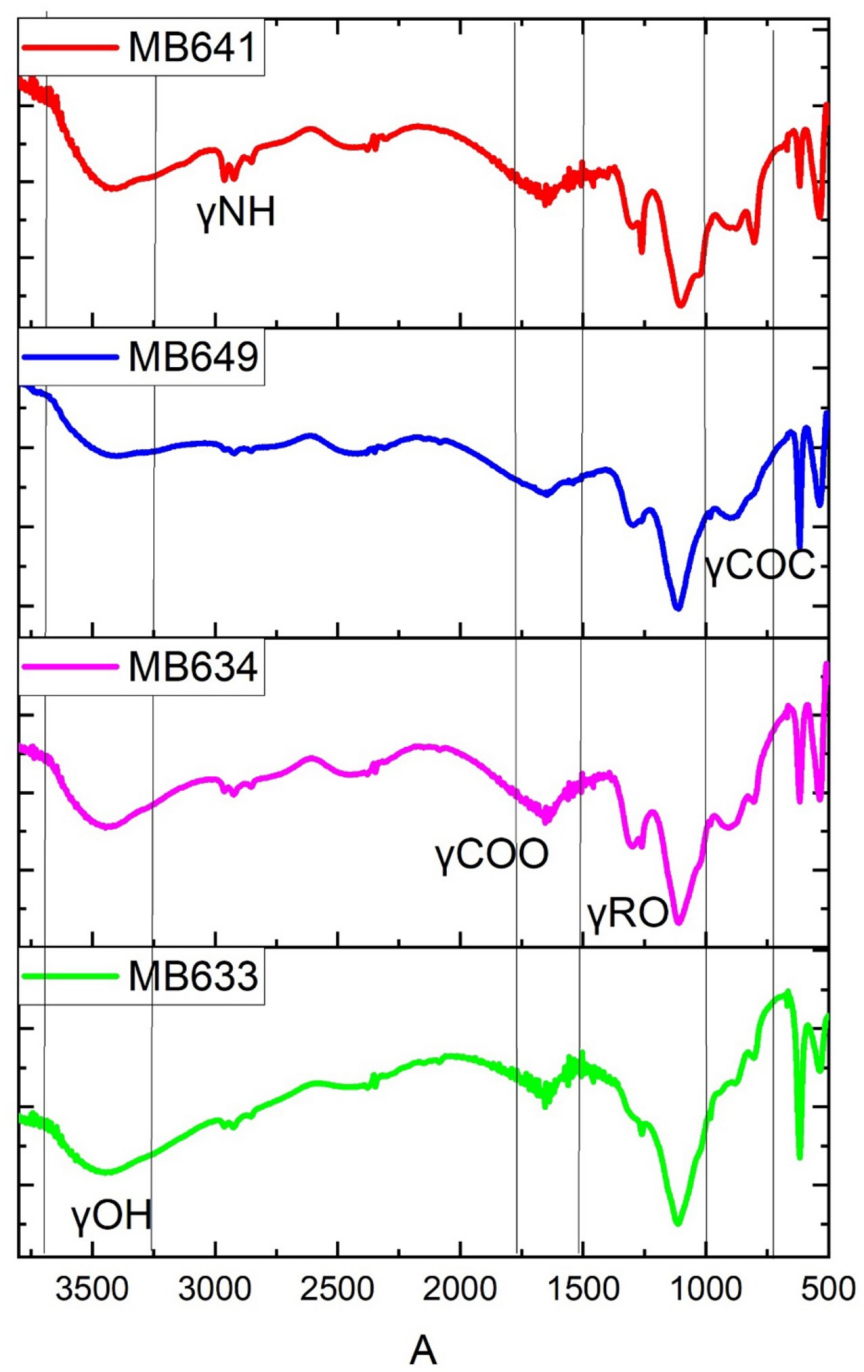
FTIR Spectra of selected EPS matrix obtained from different bacterial strains. The figure represents the Fourier transform infrared spectroscopic analysis of EPS of different pure-culture bacteria. The figure shows the major functional groups present in the EPS. Infrared spectra of bacterial EPS indicated the alcohols, Carboxylic acids, esters, alkyl halides, and alkenes functional groups. The figure is prepared in Originpro 8.5 software.

In the present study, the EPS matrix was extracted to study different functional groups present in it, such as protein, humic substances, and polysaccharides, along with other substances. The position and number of FTIR peaks for the EPS fractions seemed to be relatively close, suggesting that the nature of chemical groups in these fractions were similar (Fig 5). Numerous strong frequency band linked with proteins and polysaccharides were readily observed in the EPS components. The results showed predominant spectral bands for hydroxyl (OH) at 3600–3200 cm^−1^, amide (NH) at 2970–2850 cm^−1^, weak peak of carbonyl (–COOR) at 1740 cm ^− 1^, alkoxyl ester (RO) at 1255 cm ^− 1^, carbonyl (C OC) at 1167 cm ^− 1^, and acetal linkage (O–C–O) at 1039 cm ^− 1^ [37, 38].

### X-ray diffraction (XRD)

**Fig 6:**
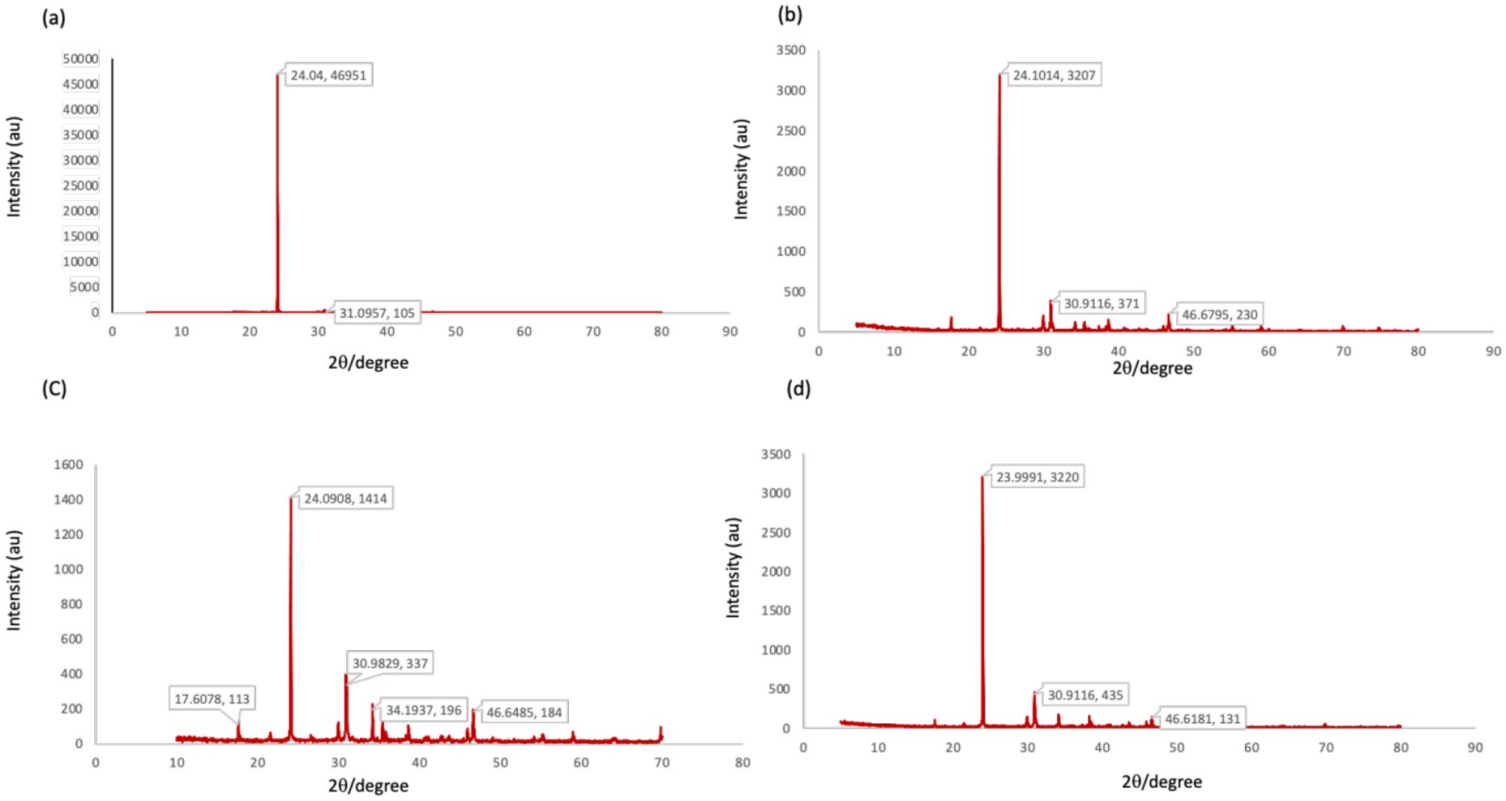
X-ray diffraction profile of EPS matrices extracted from biofilm forming strains (a) *P*. *aeruginosa* MB633, (b) *S*. *aureus* MB634, (c), MB641 *E*. *coli* and (d) *Enterobacter cloacae* MB649. Sharp, high-intensity peaks in XRD pattern typically indicate the presence of well-ordered crystalline material. The position of the peaks, represented as 2*θ* (where *θ* is the diffraction angle), corresponds to the interatomic spacing within the crystal lattice.

X-ray diffraction profile of EPS was carried out to study the nature of the extracellular polymeric substance. All the selected samples showed characteristic sharp peaks at 24.10°, 30.91° and 46.67° indicating crystalline and the well-developed nature of the compound (Fig 6).

### Biofilm formation ability

**Fig 7:**
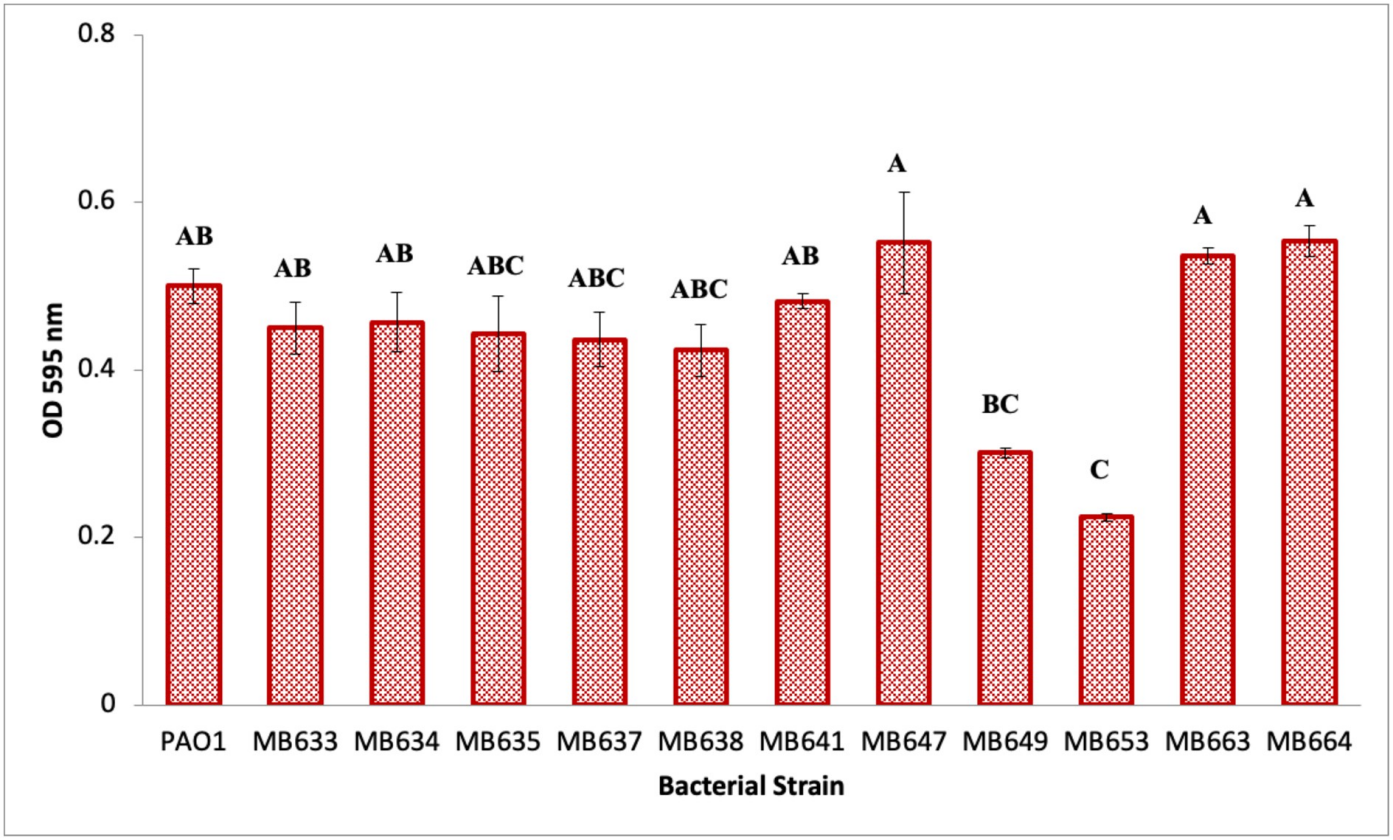
Biofilm forming ability of selected strains isolated from infected orthopaedic implants. Data in the above figure represents mean of three technical replicates ± SD. Samples with different letters represents significant values among the applied treatment. Level of significance was evaluated at the 95% confidence level by Fisher Pairwise comparison with One Way ANOVA.

PAO1 was used as a reference strain for studying biofilm producing strains. PAO1 is a well-studied strain and a known biofilm former, which makes it a good model for studying biofilm formation in other bacteria [39]. The biofilm forming ability of the identified strains was evaluated using the classic crystal violet assay after 24 hours of incubation. Within the spectrum of bacterial strains investigated, *Bacillus* spp. MB647 exhibited the highest biofilm-forming ability, with a value of 0.55 ± 0.06. Notably, *Pseudomonas* spp. strains MB663 and MB664 also demonstrated significant biofilm-forming potential, with values of 0.54 ± 0.01 and 0.52 ± 0.02 respectively, after the 24-hour incubation period. The strains that exhibited the lowest biofilm-forming abilities were *Enterobacter cloacae* MB649, with a value of 0.3 ± 0.006, and *Planococcus chinensis* MB653, which showed the lowest value of 0.22 ± 0.004. The remaining strains displayed biofilm formation within the range of 0.42 to 0.48 (as shown in Fig 7). These findings provide insights into the varying biofilm-forming capacities of the bacterial strains under study after the specified 24-hour incubation period.

Fisher pairwise comparison with one way ANOVA significantly depicted *p* = 0.000 high biofilm formation in all the tested strains except for MB653 and MB649. Both of these strains have significantly *p* < 0.05 low biofilm producing abilities which are validated by statistical analysis. Although the biofilm formation capacity of all the strains was good, the comparison between them did not reveal any significant difference. The level of significance is mentioned alphabetically in the Fig 7.

## Discussion

The microbiological diagnosis of orthopaedic implant-associated infections is vital for effective management. These infections are a major concern for patients and can lead to prolonged hospitalization, increased healthcare costs, and even the need for revision surgery, especially in developing countries [40]. This study focused on the phenotypic variation and the antibiotic resistance pattern in the causative pathogens isolated from infected orthopaedic implant samples. Its findings highlight a significant rate of antibiotic resistance in bacterial isolates obtained from 16 surgical implant infections in a one-year sampling period. Initially, bacterial isolates were identified through morphological and biochemical characterization methods. Various bacterial strains were successfully isolated and identified in this present study, such as *Staphylococcus aureus*, as well as multidrug-resistant Gram-negative rods such as *E*. *coli*, *Klebsiella pneumonia*, and *Pseudomonas aeruginosa*. According to a study by Moftian *et al*. the prevalence of antibiotic-resistant Gram-negative bacteria, such as *E*. *coli*, *Klebsiella pneumoniae*, and *Pseudomonas aeruginosa*, is highest in Asia and in the Middle East, ranging from 30% to 80% depending on the antibiotic used [41]. A study that analysed the global distribution and transmission of carbapenem-resistant *Enterobacteriaceae* (CRE) suggested that South Asia has a high frequency of carbapenem-resistant *Enterobacteriaceae* [42]. Other studies mentioned coagulase-negative *Staphylococci*, *S*. *aureus*, *Enterococcus* species and *E*.*coli* from such similar infections [43, 44]. In this current study, the antibiotic resistance pattern of the 44 clinical isolates was investigated. The MAR index was used to identify the resistance pattern in the clinical isolates. The MAR index ≥ 0.2 indicates high resistance and increased use of antibiotics in that specific area [45]. Several studies have looked into the MAR index (MARI) of bacteria obtained from samples of human tissue. In 2016, a study examined data from patients in India and discovered that the average MARI was greater than 0.2, indicating evidence of antibiotic resistance [46]. A study conducted in Pakistan showed multi-resistance in 157 (81.3%) bacterial strains, while 162 strains had a multi-antibiotic resistance index (MAR) of 0.2. [47]. Muhindo *et al*. examined patients with surgical site infections in Uganda and reported an average MARI of 0.6, signifying substantial antibiotic resistance [48]. Our study revealed a significant MAR index of 0.6. Furthermore, the results indicated that over 90% of isolates had a MAR index of 0.2. This high level of antibiotic resistance presents clinicians with the challenge of limited treatment choices. Multidrug resistance further narrows available options for antibiotic therapy [49–51].

After the initial assessment, eleven bacterial isolates were selected for identification via 16S rRNA gene sequencing based on their physiological, biochemical characterization, and antibiotic resistance profiles. Production of extracellular polymeric substance (EPS) of the selected strains was assessed and quantified. It is a well-known fact that EPS play a key role in the attachment as well as colonization of bacteria on surfaces, including implant surfaces, and also provide a physical barrier that protect the bacteria from the host immune system. It was observed by researchers that some bacterial isolates have been found to be both more hydrophobic and produce more EPS [52]. Researchers suggested that the amount of EPS required to form mature colonies by different bacteria, depend upon genus or species [53].

In the current study, maximum EPS production as well as good biofilm film forming ability and hydrophobicity was observed in all *Pseudomonas aeruginosa* strains. This finding is in line with previous research suggesting that *Pseudomonas* species require greater EPS production, particularly for primary adhesion to substrates [54]. This combination of characteristics makes them particularly well suited to form biofilms on implant surfaces and can lead to increased resistance to antibiotics which was observed in the bacterial strains isolated from the samples. These bacteria have been found to form biofilms on the implant surface, allowing them to evade the host immune response and antibiotics [55–58]. Studies have shown that EPS contribute to antibiotic resistance through various mechanisms. A key method involves limiting antibiotic penetration via the polysaccharide matrix [59]. Biofilm EPS shields bacteria from the host’s complement system, reducing immune response and promoting survival [60, 61]. Protective EPS in mixed bacterial biofilms helps non-resistant bacteria endure antibiotics and even transfer resistance genes [61]. Research shows exopolysaccharide degrading enzymes (EPDEs) enhance antibiotic efficacy, as seen with *P*. *aeruginosa* treated with ciprofloxacin and EPDE alginate lyase in mouse models [62, 63].

Various functional groups within the EPS matrix were investigated in the current research, including proteins, humic substances, polysaccharides, and other components, using FTIR spectroscopy. The analysis confirmed the presence of proteins, humic substances, and nucleic acids in the samples [64, 65]. The characteristic peaks, indicative of amide and carboxylic groups, were consistent across all EPS samples and aligned with prior studies [66, 67]. It is evident from the comparative spectra of selected samples of EPS matrices that they possess similarity in terms of their chemical composition and proportion of chemical constituents. X-ray diffraction technique was applied for phase identification of extracellular polymeric substance. The XRD diffractograms showed predominantly sharp peaks that indicate predominance of the crystallinity and uniform crystal lattice, as shown in previous literature [68]. The results are encouraging in terms of high crystallinity which gives a strong three dimensional architecture of biofilms thereby acting as a reinforcing grid to form strong multicellular entities [69, 70].

The results of the present study showed that the detection of orthopaedic implant-associated infections through microbiological testing is critical in treating complex cases and preventing further complications. For the purpose of creating efficient treatment plans, it is essential to identify the bacterial species and patterns of antibiotic resistance in implant infection samples. The significant rate of antibiotic resistance discovered in this study emphasises the urgent requirement for new, affordable medicines to treat bacterial surgical site infections. Hence, more investigation is needed to find new therapeutic modalities that can supplement or lessen the need of antibiotics. In addition to helping patients, this will also contribute to addressing the growing issue of antibiotic resistance in hospital settings.

## Conclusion

Antibiotic resistance has become a global issue and is causing high mortality rates worldwide. This study indicates that the menace of MDR pathogens is prevalent in Pakistan because of the indiscriminate and excessive use of antibiotics without appropriate investigation and physician’s knowledge. It is a well-known fact that the introduction of a foreign object into its host, such as an orthopaedic device, would increase the susceptibility of infection. The management of these orthopaedic device-associated infections represents a great challenge to health practitioners and researchers. The results of this study provides better insight in understanding the role of various virulence factors that may have a role in the colonization and the adaptation of the etiological agents in the host.

## Supporting information

S1 Table Morphological characters of bacteria obtained from infected implants. (DOCX)

S2 Table Cell morphology of bacterial isolates obtained from infected implants. (DOCX)

S3 Table Biochemical characters of the bacterial isolates obtained from infected orthopaedic implant samples. (DOCX)

S4 Table Biochemical characters of the bacterial isolates obtained from infected orthopaedic implant samples. (DOCX)

## References

[1] Shahid A, Aslam B, Muzammil S, Aslam N, Shahid M, Almatroudi A, et al. The prospects of antimicrobial coated medical implants. Journal of applied biomaterials and functional materials. 2021; 19:22808000211040304. doi: 10.1177/22808000211040304 34409896

[2] Syeddan SA. Research methodology and mechanisms of action of current orthopaedic implant coatings. Journal of Long-Term Effects of Medical Implants. 2023; 33(2). doi: 10.1615/JLongTermEffMedImplants.2022040062 36734927

[3] Connaughton A, Childs A, Dylewski S, Sabesan VJ. Biofilm disrupting technology for orthopedic implants: what’s on the horizon?. Frontiers in medicine. 2014; 15:1:22. doi: 10.3389/fmed.2014.00022 25705632 PMC4335381

[4] Porrino J, Wang A, Moats A, Mulcahy H, Kani K. Prosthetic joint infections: diagnosis, management, and complications of the two-stage replacement arthroplasty. Skeletal Radiology. 2020; 49:847–59. doi: 10.1007/s00256-020-03389-w 32040604

[5] Ronin D, Boyer J, Alban N, Natoli RM, Johnson A, Kjellerup BV. Current and novel diagnostics for orthopedic implant biofilm infections: a review. APMIS. 2022; 130(2):59–81. doi: 10.1111/apm.13197 34862649

[6] Shahi A, Parvizi J. Prevention of periprosthetic joint infection. Archives of bone and joint surgery. 2015; 2:72. PMC4468618 26110171

[7] Seebach E, Kubatzky KF. Chronic implant-related bone infections—can immune modulation be a therapeutic strategy?. Frontiers in immunology. 2019; 10:1724. doi: 10.3389/fimmu.2019.01724 31396229 PMC6664079

[8] De Simone B, Sartelli M, Coccolini F, Ball CG, Brambillasca P, Chiarugi M, et al. Intraoperative surgical site infection control and prevention: a position paper and future addendum to WSES intra-abdominal infections guidelines. World journal of emergency surgery. 2020; 15(1):1–23. doi: 10.1186/s13017-020-0288-4 32041636 PMC7158095

[9] McGarty TP. Biofilm Growth and infiltration.2016. doi: 10.13140/RG.2.1.3319.3846

[10] Li P, Gao Z, Tan Z, Xiao J, Wei L, Chen Y. New developments in anti-biofilm intervention towards effective management of orthopedic device related infections (ODRI’s). Biofouling. 2020; 37(1):1–35. 10.1080/08927014.2020.1869725 33618584

[11] Coppola GA, Onsea J, Moriarty TF, Nehrbass D, Constant C, Zeiter S, et al. An improved 2-aminoimidazole based anti-biofilm coating for orthopedic implants: activity, stability, and in vivo Biocompatibility. Frontiers in Microbiology. 2021; 12:658521. doi: 10.3389/fmicb.2021.658521 33967997 PMC8097006

[12] Arciola CR, Campoccia D, Montanaro L. Implant infections: adhesion, biofilm formation and immune evasion. Nature reviews microbiology. 2018; 16(7):397–409. doi: 10.1038/s41579-018-0019-y 29720707

[13] Wang K, Li W, Liu H, Yang Y, Lv L. Progress in prevention, diagnosis, and treatment of periprosthetic joint infection. evidence-based complementary and alternative medicine. 2021; 2021. doi: 10.1155/2021/3023047 33542741 PMC7840269

[14] Iskandar K, Murugaiyan J, Hammoudi Halat D, Hage SE, Chibabhai V, et al. Antibiotic discovery and resistance: the chase and the race. Antibiotics. 2022; 11(2):182. doi: 10.3390/antibiotics11020182 35203785 PMC8868473

[15] Roy S, Chowdhury G, Mukhopadhyay AK, Dutta S, Basu S. Convergence of biofilm formation and antibiotic resistance in *Acinetobacter baumannii* infection. Frontiers in medicine. 2022; 9:793615. 35402433 10.3389/fmed.2022.793615 PMC8987773

[16] Moriarty TF, Kuehl R, Coenye T, Metsemakers WJ, Morgenstern M, Schwarz EM, et al. Orthopaedic device-related infection: current and future interventions for improved prevention and treatment. EFORT open reviews. 2016; 1(4):89–99. doi: 10.1302/2058-5241.1.000037 28461934 PMC5367564

[17] Kumar A, Rai A. Prevalence of surgical site infection in general surgery in a tertiary care centre in India. International Surgery Journal. 2017; 4(9):3101–6.

[18] Jun Y, Jianghua L. Diagnosis of periprosthetic joint infection using polymerase chain reaction: an updated systematic review and meta-analysis. Surgical infections. 2018; 19(6):555–65. doi: 10.1089/sur.2018.014 29920159

[19] Aamot HV, Johnsen BO, Skramm I. Rapid diagnostics of orthopedic implant-associated infections using Unyvero ITI implant and tissue infection application is not optimal for *Staphylococcus* species identification. BMC Research Notes. 2019; 12:1–8. 31694724 10.1186/s13104-019-4755-5 PMC6836655

[20] Köder K, Hardt S, Gellert MS, Haupenthal J, Renz N, Putzier M, et al. Outcome of spinal implant-associated infections treated with or without biofilm-active antibiotics: results from a 10-year cohort study. Infection. 2020; 48:559–68. doi: 10.1007/s15010-020-01435-2 32372396 PMC7395063

[21] Pfang BG, García-Cañete J, García-Lasheras J, Blanco A, Auñón Á, Parron-Cambero R, et al. Orthopedic implant-associated infection by multidrug resistant *Enterobacteriaceae*. Journal of Clinical Medicine. 2019; 8(2):220. 30744054 10.3390/jcm8020220 PMC6406851

[22] Coraça-Huber DC, Kreidl L, Steixner S, Hinz M, Dammerer D, Fille M. Identification, and morphological characterization of biofilms formed by strains causing infection in orthopedic implants. Pathogens. 2020; 9(8):649. doi: 10.3390/pathogens9080649 32806685 PMC7460306

[23] Alelign D, Tena T, Tadesse D, Tessema M, Seid M, Oumer Y, et al. Bacteriological profiles, antimicrobial susceptibility patterns, and associated factors in patients undergoing orthopedic surgery with suspicion of surgical site infection at Arba Minch General Hospital in Southern Ethiopia. Infection and Drug Resistance. 2022; 1:2427–43. doi: 10.2147/IDR.S367510 35592104 PMC9112451

[24] Singh A, Singh RK, Bimal BK, Runu R. Type of microbial flora in patients with bone and joint infections: Our experience at a tertiary care center of Eastern India. Journal of Orthopaedic Diseases and Traumatology. 2023; 6(1): 58–61.

[25] Afshan N, Ullah SK, Kazmi M. Evaluation of indigenously developed quick identification system (QTS-12) with API 10S. In 2018 15^th^ International Bhurban Conference on Applied Sciences and Technology (IBCAST). 2018; 218–221.

[26] Bauer AW, Kirby WM, Sherris JC, Turck M. Antibiotic susceptibility testing by a standardized single disk method. American journal of clinical pathology. 1966; 1(45): 493–6. 5325707

[27] Hombach M, Bloemberg GV, Böttger EC. Effects of clinical breakpoint changes in CLSI guidelines 2010/2011 and EUCAST guidelines 2011 on antibiotic susceptibility test reporting of Gram-negative *bacilli*. Journal of antimicrobial chemotherapy. 2012; 67(3):622–32. 22167240 10.1093/jac/dkr524

[28] Kameoka S, Motooka D, Watanabe S, Kubo R, Jung N, Midorikawa Y, et al. Benchmark of 16S rRNA gene amplicon sequencing using Japanese gut microbiome data from the V1–V2 and V3–V4 primer sets. BMC genomics. 2021; 22(1):1–0. 34246242 10.1186/s12864-021-07746-4 PMC8272389

[29] Vingataramin L, Frost EH. A single protocol for extraction of gDNA from bacteria and yeast. Biotechniques. 2015; 58(3):120–5. doi: 10.2144/000114263 25757544

[30] Verhoef R, De Waard P, Schols HA, Siika-aho M, Voragen AG. *Methylobacterium* sp. isolated from a Finnish paper machine produces highly pyruvated galactan exopolysaccharide. Carbohydrate Research. 2003; 338(18):1851–9. 12932368 10.1016/s0008-6215(03)00261-1

[31] Bradford MM. A rapid and sensitive method for the quantitation of microgram quantities of protein utilizing the principle of protein-dye binding. Analytical biochemistry. 1976; 72: 248–54. doi: 10.1006/abio.1976.9999 942051

[32] DuBois M, Gilles KA, Hamilton JK, Rebers PT, Smith F. Colorimetric method for determination of sugars and related substances. Analytical chemistry. 1956; 28(3):350–6.

[33] Tahir U, Yasmin A. Decolorization and discovery of metabolic pathway for the degradation of Mordant Black 11 dye by *Klebsiella* sp. MB398. Brazilian Journal of Microbiology. 2021; 52:761–71. 33754316 10.1007/s42770-021-00470-x PMC8105450

[34] Kavita K, Mishra A, Jha B. Isolation, and physico-chemical characterisation of extracellular polymeric substances produced by the marine bacterium Vibrio parahaemolyticus. Biofouling. 2011; 27(3):309–17. doi: 10.1080/08927014.2011.562605 21409653

[35] Chao Y, Guo F, Fang HH, Zhang T. Hydrophobicity of diverse bacterial populations in activated sludge and biofilm revealed by microbial adhesion to hydrocarbons assay and high-throughput sequencing. Colloids and Surfaces B: Biointerfaces. 2014; 114:379–85. doi: 10.1016/j.colsurfb.2013.10.028 24246196

[36] O’Toole GA. Microtiter dish biofilm formation assay. JoVE. (Journal of Visualized Experiments). 2011; 47: e2437.47. doi: 10.3791/2437 21307833 PMC3182663

[37] Casarin J, Gonçalves AC Jr, Segatelli MG, Tarley CR. Poly (methacrylic acid)/SiO2/Al2O3 based organic-inorganic hybrid adsorbent for adsorption of imazethapyr herbicide from aqueous medium. Reactive and Functional Polymers. 2017; 121:101–9.

[38] Asif I, Rafique U. Synthesis & fabrication of O-linked polymeric hybrids for recovery of textile dyes: Closed loop economy. Environmental Research. 2023; 116780. 10.1016/j.envres.2023.116780 37527750

[39] Lima JL, Alves LR, Jacomé PR, Bezerra Neto JP, Maciel MA, et al. Biofilm production by clinical isolates of *Pseudomonas aeruginosa* and structural changes in LasR protein of isolates non-biofilm producing. Brazilian Journal of Infectious Diseases. 2018; 22:129–36. 10.1016/j.bjid.2018.03.003 PMC9428190 29601791

[40] Ayukekbong JA, Ntemgwa M, Atabe AN. The threat of antimicrobial resistance in developing countries: causes and control strategies. Antimicrobial Resistance and Infection Control. 2017; 6(1):1–8. 10.1186/s13756-017-0208-x PMC5433038 28515903

[41] Moftian N, Rezaei-Hachesu P, Arab-Zozani M, Samad-Soltani T, Esfandiari A, Tabib MS, et al. Prevalence of gram-negative bacteria and their antibiotic resistance in neonatal sepsis in Iran: a systematic review and meta-analysis. BMC Infectious Diseases. 2023; 23(1):1–5. 37582726 10.1186/s12879-023-08508-1 PMC10426195

[42] Logan LK, Weinstein RA. The epidemiology of carbapenem-resistant *Enterobacteriaceae*: the impact and evolution of a global menace. Journal of Infection and Public Health. 2017; 10(4), 404–417. 10.1093/infdis/jiw282 PMC5853342 28375512

[43] Wu D, Ding Y, Yao K, Gao W, Wang Y. Antimicrobial resistance analysis of clinical Escherichia coli isolates in neonatal ward. Frontiers in pediatrics. 2021; 9:670470. doi: 10.3389/fped.2021.670470 34113589 PMC8185016

[44] Roohi S, Ahmed T, Altaf I, Fomda B. Isolation of Methicillin-Resistant *Staphylococcus aureus* from Wound Samples during the COVID-19 Pandemic: A Retrospective Study. Journal of Clinical & Diagnostic Research. 2023; 17(3).

[45] Ayandele AA, Oladipo EK, Oyebisi O, Kaka MO. Prevalence of multi-antibiotic resistant *Escherichia coli* and *Klebsiella* species obtained from a tertiary medical institution in Oyo State, Nigeria. Qatar medical journal. 2020.;1:9. 10.5339/qmj.2020.9 PMC7118460 32280610

[46] Sandhu R, Dahiya S, Sayal P. Evaluation of multiple antibiotic resistance (MAR) index and Doxycycline susceptibility of Acinetobacter species among inpatients. Indian J. Microbiol. Res. 2016; 3(3):299

[47] Imran H, Khan Z, Saleem F, Gull S, Tahir A. The growing threat of antibiotic resistance in wound infections: Evidence from tertiary care in Pakistan. Archives of Biological Sciences. 2023; (00):21–21.

[48] Muhindo AB, Aliero AA, Odoki M, Ntulume I, Eilu E, Mutebi J, et al. Antibiotic-Resistant Profiles of Bacteria Isolated from Cesarean and Surgical Patients from Kasese District Hospitals Western Uganda. 2021.

[49] Riaz S, Faisal M, Hasnain S. Antibiotic susceptibility pattern and multiple antibiotic resistances (MAR) calculation of extended spectrum β-lactamase (ESBL) producing *Escherichia coli* and *Klebsiella* species in Pakistan. African Journal of Biotechnology. 2011;10(33):6325–31.

[50] Kourtis AP, Sheriff EA, Weiner-Lastinger LM, Elmore K, Preston LE, Dudeck M, et al. Antibiotic multidrug resistance of *Escherichia coli* causing device-and procedure-related infections in the United States reported to the National Healthcare Safety Network, 2013–2017. Clinical Infectious Diseases. 2021; 73(11):e4552–9. 32702102 10.1093/cid/ciaa1031 PMC9377353

[51] Saliba R, Zahar JR, Dabar G, Riachy M, Karam-Sarkis D, Husni R. Limiting the spread of multidrug-resistant bacteria in low-to-middle-income countries: one size does not fit all. Pathogens. 2023; 12(1):144. doi: 10.3390/pathogens12010144 36678492 PMC9866331

[52] Vu B, Chen M, Crawford RJ, Ivanova EP. Bacterial extracellular polysaccharides involved in biofilm formation. Molecules. 2009; 14(7):2535–54. doi: 10.3390/molecules14072535 19633622 PMC6254922

[53] Muhammad MH, Idris AL, Fan X, Guo Y, Yu Y, Jin X, et al. Beyond risk: bacterial biofilms and their regulating approaches. Frontiers in microbiology. 2020; 11:928. doi: 10.3389/fmicb.2020.00928 32508772 PMC7253578

[54] Whitchurch CB, Tolker-Nielsen T, Ragas PC, Mattick JS. Extracellular DNA required for bacterial biofilm formation. Science. 2002; 295(5559):1487–1487. doi: 10.1126/science.295.5559.1487 11859186

[55] Bilal H, Khan MN, Rehman T, Hameed MF, Yang X. Antibiotic resistance in Pakistan: a systematic review of past decade. BMC infectious diseases. 2021; 21(1):1–9. 33676421 10.1186/s12879-021-05906-1 PMC7937258

[56] Lu Y, Cai WJ, Ren Z, Han P. The role of *Staphylococcal* biofilm on the surface of implants in orthopedic infection. Microorganisms. 2022; 10(10):1909. 36296183 10.3390/microorganisms10101909 PMC9612000

[57] Iqbal S, Bhatti SM, Amir M, Zaman QU, Raza A. Scenario of Antibiotic Resistance in Pakistan: A Systematic Review. Pakistan Journal of Medical and Health Sciences. 2022; 16(05):643–643.

[58] Israr F, Hassan F, Ali H, Hasan SM, Jabeen S, Israr Z. Antibacterial and physicochemical properties of local and international brands of moxifloxacin used in clinical patient care in Pakistan. RADS Journal of Pharmacy and Allied Health Sciences. 2023; 1(1):16–22.

[59] Yasir M, Willcox MD, Dutta D. Action of antimicrobial peptides against bacterial biofilms. Materials. 2018; 11(12):2468. doi: 10.3390/ma11122468 30563067 PMC6317029

[60] Yan J, Bassler BL. Surviving as a community: antibiotic tolerance and persistence in bacterial biofilms. Cell host and microbe. 2019; 26(1):15–21. doi: 10.1016/j.chom.2019.06.002 31295420 PMC6629468

[61] Singh S, Datta S, Narayanan KB, Rajnish KN. Bacterial exo-polysaccharides in biofilms: Role in antimicrobial resistance and treatments. Journal of Genetic Engineering and Biotechnology. 2021; 19(1):1–9. 34557983 10.1186/s43141-021-00242-y PMC8460681

[62] Alipour M, Suntres ZE, Omri A. Importance of DNase and alginate lyase for enhancing free and liposome encapsulated aminoglycoside activity against *Pseudomonas aeruginosa*. Journal of Antimicrobial Chemotherapy. 2009; 64(2):317–25. 19465435 10.1093/jac/dkp165

[63] Blanco-Cabra N, Paetzold B, Ferrar T, Mazzolini R, Torrents E, Serrano L, et al. Characterization of different alginate lyases for dissolving *Pseudomonas aeruginosa* biofilms. Scientific Reports. 2020; 10(1):9390. 32523130 10.1038/s41598-020-66293-2 PMC7287115

[64] Jiao Y, Cody GD, Harding AK, Wilmes P, Schrenk M, Wheeler KE, et al. Characterization of extracellular polymeric substances from acidophilic microbial biofilms. Applied and environmental microbiology. 2010; 76(9):2916–22. doi: 10.1128/AEM.02289-09 20228116 PMC2863431

[65] Dutta Sinha S, Chatterjee S, Maiti PK, Tarafdar S, Moulik SP. Evaluation of the role of substrate and albumin on *Pseudomonas aeruginosa* biofilm morphology through FESEM and FTIR studies on polymeric biomaterials. Progress in biomaterials. 2017; 6:27–38. 28155216 10.1007/s40204-017-0061-2 PMC5433955

[66] Cheng H, Liu H, Shi Z, Xu Y, Lian Q, Zhong Q, et al. Long-term antibacterial and biofilm dispersion activity of an injectable in situ crosslinked co-delivery hydrogel/microgel for treatment of implant infection. Chemical Engineering Journal. 2022; 433:134451.

[67] Sinha SD, Choudhuri M, Basu T, Gupta D, Datta A. Decisive role of polymer–bovine serum albumin interactions in biofilm substrates on “philicity” and extracellular polymeric substances composition. Langmuir. 2022; 38(6):1966–76. doi: 10.1021/acs.langmuir.1c00187 35119288

[68] Ribeiro PL, Campos MI, Druzian JI. Novel extracellular polymeric substances produced by Cupriavidus necator IPT 027 grown on glucose and crude glycerol originated from biodiesel. Polymers for Advanced Technologies. 2017; 28(4):549–56.

[69] Mandal SK, Singh RP, Patel V. Isolation and characterization of exopolysaccharide secreted by a toxic dinoflagellate, Amphidinium carterae Hulburt 1957 and its probable role in harmful algal blooms (HABs). Microbial ecology. 2011; 62:518–27. doi: 10.1007/s00248-011-9852-5 21503775

[70] Padmavathi AR, Periyasamy M, Pandian SK. Assessment of 2, 4-di-tert-butylphenol induced modifications in extracellular polymeric substances of *Serratia marcescens*. Bioresource Technology. 2015; 188:185–9. 25641715 10.1016/j.biortech.2015.01.049

