# Supplementary material for: Insights into the microbiological and virulence characteristics of bacteria in orthopaedic implant infections: A study from Pakistan
Source: PLoS One. 2023 Oct 17;18(10):e0292956. doi: 10.1371/journal.pone.0292956 (PMC10581495; doi:10.1371/journal.pone.0292956)
Supplement: S1 Table — (DOCX) [file pone.0292956.s001.docx]

**Supplementary information**

**S1 Table.** Morphological characters of bacteria obtained from infected implants.

| **Bacterial**  **Isolates** | **Shape** | **Visual color** | **Color under microscope** | **Elevation** | **Margin** | **Texture** | **Size (mm)** |
| --- | --- | --- | --- | --- | --- | --- | --- |
| **MB631** | Circular | OW | Brown | Raised | Smooth | Creamy | 3-4 |
| **MB632** | Circular | OW | Brown | Raised | Smooth | Creamy | 1.5-2 |
| **MB633** | Circular | G | Medium Brown | Flat | Smooth | Mucilaginous | 1-2 |
| **MB634** | Circular | Y | Brown | Convex | Smooth | Creamy | 1-2 |
| **MB635** | Circular | G | Brown | Flat | Smooth | Mucilaginous | 1-2 |
| **MB636** | Circular | OW | Brown | Raised | Smooth | Watery | 2-3 |
| **MB637** | Circular | G | Brown | Flat | Smooth | Mucilaginous | 1.5-2 |
| **MB638** | Circular | G | Brown | Raised | Smooth | Mucilaginous | 1-2 |
| **MB639** | Circular | Y | Brown | Convex | Smooth | Creamy | 1-2 |
| **MB640** | Circular | OW | Brown | Slightly raised | Smooth | Creamy | 2 |
| **MB641** | Circular | OW | Brown | Slightly raised | Smooth | Creamy | 2 |
| **MB642** | Circular | OW | Brown | Slightly raised | Smooth | Creamy | 2 |
| **MB643** | Circular | OW | Brown | Slightly raised | Smooth | Creamy | 2 |
| **MB644** | Circular | OW | Brown | Slightly convex | Smooth | Creamy | 1.5 |
| **MB645** | Circular | OW | Brown | Slightly raised | Smooth | Creamy | 1.5 |
| **MB646** | Circular | OW | Brown | Slightly raised | Smooth | Creamy | 1.5 |
| **MB647** | Wrinkled | OW | Brown | Slightly raised | Irregular | Pasty | 2-3 |
| **MB648** | Circular | OW | Brown | Slightly raised | Smooth | Creamy | 2 |
| **MB649** | Circular | OW | Brown | Raised | Smooth | Creamy | 1-2 |
| **MB650** | Circular | W | Brown | Convex | Smooth | Creamy | 1-1.5 |
| **MB651** | Circular | OW | Brown | Raised | Smooth | Creamy | 1-2 |
| **MB652** | Circular | OW | Brown | Raised | Smooth | Creamy | 1-2 |
| **MB653** | Circular | O | Brown | Raised | Smooth | Creamy | 0.5-1 |
| **MB654** | Circular | OW | Brown | Raised | Smooth | Creamy | 1-2 |
| **MB655** | Circular | OW | Brown | Raised | Smooth | Creamy | 1-2 |
| **MB656** | Circular | Y | Brown | Convex | Smooth | Creamy | 1.5 |
| **MB657** | Circular | Y | Brown | Convex | Smooth | Creamy | 1-2 |
| **MB658** | Circular | Y | Brown | Convex | Smooth | Creamy | 1-2 |
| **MB659** | Circular | Y | Brown | Convex | Smooth | Creamy | 1.5 |
| **MB660** | Circular | Y | Brown | Raised | Smooth | Creamy | 1-2 |
| **MB661** | Circular | Y | Brown | Convex | Smooth | Creamy | 1-2 |
| **MB662** | Circular | Y | Brown | Convex | Smooth | Creamy | 1-2 |
| **MB663** | Circular | G | Brown | Raised | Smooth | Mucilaginous | 1 |
| **MB664** | Circular | G | Brown | Raised | Smooth | Mucilaginous | 1 |
| **MB665** | Circular | G | Brown | Raised | Smooth | Mucilaginous | 1.5 |
| **MB666** | Circular | G | Brown | Raised | Smooth | Mucilaginous | 1 |
| **MB667** | Circular | G | Brown | Raised | Smooth | Mucilaginous | 1 |
| **MB668** | Circular | G | Brown | Raised | Smooth | Mucilaginous | 1.5 |
| **MB669** | Circular | Y | Brown | Convex | Smooth | Creamy | 1-2 |
| **MB670** | Circular | Y | Brown | Convex | Smooth | Creamy | 1-2 |
| **MB671** | Circular | Y | Brown | Convex | Smooth | Creamy | 1.5 |
| **MB672** | Circular | Y | Brown | Convex | Smooth | Creamy | 1.5 |
| **MB673** | Circular | Y | Brown | Convex | Smooth | Creamy | 1-2 |
| **MB674** | Circular | Y | Brown | Convex | Smooth | Creamy | 1-2 |

OW= Off-white, G=Greenish, Y= Yellow, W=White, O=Orange
