# Supplementary material for: Insights into the microbiological and virulence characteristics of bacteria in orthopaedic implant infections: A study from Pakistan
Source: PLoS One. 2023 Oct 17;18(10):e0292956. doi: 10.1371/journal.pone.0292956 (PMC10581495; doi:10.1371/journal.pone.0292956)
Supplement: S2 Table — (DOCX) [file pone.0292956.s002.docx]

**S2 Table.** Cell morphology of bacterial isolates obtained from infected implants.

| **Bacterial**  **Isolates** | **Gram Straining** | **Shape** | **Arrangement** | **Capsule Staining** |
| --- | --- | --- | --- | --- |
| **MB631** | G-ve | Rods | Single cell | + |
| **MB632** | G+ve | Rods | Single, pairs | + |
| **MB633** | G+ve | Rods | Pairs, clusters | + |
| **MB634** | G+ve | Cocci | Clusters | - |
| **MB635** | G-ve | Rods | Pairs | + |
| **MB636** | G-ve | Cocci | Clusters | + |
| **MB637** | G-ve | Rods | Single | + |
| **MB638** | G-ve | Rods | Pairs, clusters | + |
| **MB639** | G+ve | Cocci | Cluster | + |
| **MB640** | G-ve | Rods | Single | - |
| **MB641** | G-ve | Rods | Single | - |
| **MB642** | G-ve | Rods | Single, pairs | + |
| **MB643** | G-ve | Rods | Single, pairs | + |
| **MB644** | G+ve | Rods | Filamentous | + |
| **MB645** | G+ve | Rods | Filamentous | + |
| **MB646** | G-ve | Rods | Single | + |
| **MB647** | G+ve | Rods | Filamentous | + |
| **MB648** | G-ve | Rods | Pairs | + |
| **MB649** | G-ve | Rods | Single, pairs | + |
| **MB650** | G+ve | Cocci | Cluster | + |
| **MB651** | G-ve | Rods | Pairs, clusters | + |
| **MB652** | G-ve | Rods | Single, pairs | + |
| **MB653** | G+ve | Cocci | Pairs | + |
| **MB654** | G-ve | Cocci | Cluster | + |
| **MB655** | G-ve | Rods | Pairs | + |
| **MB656** | G+ve | Cocci | Clusters | + |
| **MB657** | G+ve | Cocci | Clusters | + |
| **MB658** | G+ve | Cocci | Clusters | + |
| **MB659** | G+ve | Cocci | Clusters | + |
| **MB660** | G+ve | Rods | Pairs | + |
| **MB661** | G+ve | Cocci | Clusters | + |
| **MB662** | G+ve | Cocci | Clusters | + |
| **MB663** | G-ve | Rods | Single | + |
| **MB664** | G-ve | Rods | Single | + |
| **MB665** | G-ve | Rods | Single, pairs | + |
| **MB666** | G-ve | Rods | Single, pairs | + |
| **MB667** | G-ve | Rods | Single | + |
| **MB668** | G-ve | Rods | Single | + |
| **MB669** | G+ve | Cocci | Clusters | + |
| **MB670** | G+ve | Cocci | Clusters | + |
| **MB671** | G+ve | Cocci | Clusters | + |
| **MB672** | G+ve | Cocci | Clusters | + |
| **MB673** | G+ve | Cocci | Clusters | + |
| **MB674** | G+ve | Cocci | Clusters | + |
