# Supplementary material for: Insights into the microbiological and virulence characteristics of bacteria in orthopaedic implant infections: A study from Pakistan
Source: PLoS One. 2023 Oct 17;18(10):e0292956. doi: 10.1371/journal.pone.0292956 (PMC10581495; doi:10.1371/journal.pone.0292956)
Supplement: S4 Table — (DOCX) [file pone.0292956.s004.docx]

**S4 Table.** Biochemical characters of the bacterial isolates obtained from infected orthopaedic implant samples.

| **Bacterial**  **Isolates** | **TDA** | **IND** | **OX** | **NO_2_** | **Catalase** | **DNase** | **Coagulase** |
| --- | --- | --- | --- | --- | --- | --- | --- |
| **MB631** | 0 | 1 | 0 | 1 | 0 | 0 | 0 |
| **MB632** | 0 | 0 | 1 | 0 | 1 | 0 | 0 |
| **MB633** | 0 | 0 | 1 | 0 | 0 | 0 | 0 |
| **MB634** | 0 | 0 | 0 | 0 | 1 | 1 | 1 |
| **MB635** | 0 | 0 | 1 | 0 | 0 | 0 | 0 |
| **MB636** | 0 | 1 | 0 | 1 | 0 | 0 | 0 |
| **MB637** | 0 | 0 | 1 | 0 | 0 | 0 | 0 |
| **MB638** | 0 | 0 | 1 | 0 | 0 | 0 | 0 |
| **MB639** | 0 | 0 | 0 | 0 | 1 | 1 | 1 |
| **MB640** | 0 | 1 | 0 | 1 | 0 | 0 | 0 |
| **MB641** | 0 | 1 | 0 | 1 | 0 | 0 | 0 |
| **MB642** | 0 | 1 | 0 | 1 | 0 | 0 | 0 |
| **MB643** | 0 | 1 | 0 | 1 | 0 | 0 | 0 |
| **MB644** | 0 | 0 | 0 | 1 | 1 | 0 | 0 |
| **MB645** | 0 | 0 | 1 | 1 | 1 | 0 | 0 |
| **MB646** | 0 | 1 | 0 | 1 | 0 | 0 | 0 |
| **MB647** | 0 | 0 | 1 | 1 | 1 | 0 | 0 |
| **MB648** | 0 | 1 | 0 | 1 | 0 | 0 | 0 |
| **MB649** | 0 | 1 | 0 | 1 | 0 | 0 | 0 |
| **MB650** | 0 | 0 | 0 | 0 | 1 | 1 | 1 |
| **MB651** | 0 | 0 | 1 | 0 | 0 | 0 | 0 |
| **MB652** | 0 | 1 | 0 | 1 | 0 | 0 | 0 |
| **MB653** | 0 | 0 | 1 | 0 | 0 | 0 | 0 |
| **MB654** | 0 | 0 | 0 | 0 | 1 | 1 | 1 |
| **MB655** | 0 | 0 | 0 | 1 | 0 | 0 | 0 |
| **MB656** | 0 | 0 | 0 | 0 | 1 | 1 | 1 |
| **MB657** | 0 | 0 | 0 | 0 | 1 | 1 | 1 |
| **MB658** | 0 | 0 | 0 | 0 | 1 | 1 | 1 |
| **MB659** | 0 | 0 | 0 | 0 | 1 | 1 | 1 |
| **MB660** | 0 | 0 | 1 | 0 | 0 | 0 | 0 |
| **MB661** | 0 | 0 | 0 | 0 | 1 | 1 | 1 |
| **MB662** | 0 | 0 | 0 | 0 | 1 | 1 | 1 |
| **MB663** | 0 | 0 | 1 | 0 | 0 | 0 | 0 |
| **MB664** | 0 | 0 | 1 | 0 | 0 | 0 | 0 |
| **MB665** | 0 | 0 | 1 | 0 | 0 | 0 | 0 |
| **MB666** | 0 | 0 | 1 | 0 | 0 | 0 | 0 |
| **MB667** | 0 | 0 | 1 | 0 | 0 | 0 | 0 |
| **MB668** | 0 | 0 | 1 | 0 | 0 | 0 | 0 |
| **MB669** | 0 | 0 | 0 | 0 | 1 | 1 | 1 |
| **MB670** | 0 | 0 | 0 | 0 | 1 | 1 | 1 |
| **MB671** | 0 | 0 | 0 | 0 | 1 | 1 | 1 |
| **MB672** | 0 | 0 | 0 | 0 | 1 | 1 | 1 |
| **MB673** | 0 | 0 | 0 | 0 | 1 | 1 | 1 |
| **MB674** | 0 | 0 | 0 | 0 | 1 | 1 | 1 |

NOTE

| **TDA** | Tryptophan deaminase |
| --- | --- |
| **IND** | Indole production |
| **OX** | Oxidase |
| **NO_2_** | Nitrate reduction |

0= negative; 1= positive
